# Hydrothermal Fabrication of Highly Porous Titanium Bio-Scaffold with a Load-Bearable Property

**DOI:** 10.3390/ma10070726

**Published:** 2017-06-30

**Authors:** Han Lee, Jiunn-Der Liao, Kundan Sivashanmugan, Bernard Hao-Chih Liu, Yu-Han Su, Chih-Kai Yao, Yung-Der Juang

**Affiliations:** 1 Department of Materials Science and Engineering, National Cheng Kung University, 1 University Road, Tainan 701, Taiwan; rick594007@hotmail.com (H.L.); sivashanmugannst87@gmail.com (K.S.); hcliu@mail.ncku.edu.tw (B.H.-C.L.); kathy113kimo@yahoo.com.tw (Y.-H.S.); elleryzk@gmail.com (C.K.Y.); 2 Medical Device Innovation Center, National Cheng Kung University, 1 University Road, Tainan 701, Taiwan; 3 Department of Materials Science, National University of Tainan, Tainan 700, Taiwan; juang@mail.nutn.edu.tw

**Keywords:** porous titanium, biomimetic scaffold, load bearable, cell affinity

## Abstract

Porous titanium (P_Ti) is considered as an effective material for bone scaffold to achieve a stiffness reduction. Herein, biomimetic (bio-)scaffolds were made of sintered P_Ti, which used NaCl as the space holder and had it removed via the hydrothermal method. X-ray diffraction results showed that the subsequent sintering temperature of 1000 °C was the optimized temperature for preparing P_Ti. The compressive strength of P_Ti was measured using a compression test, which revealed an excellent load-bearing ability of above 70 MPa for that with an addition of 50 wt % NaCl (P_Ti_50). The nano-hardness of P_Ti, tested upon their solid surface, was presumably consistent with the density of pores vis-à-vis the addition of NaCl. Overall, a load-bearable P_Ti with a highly porous structure (e.g., P_Ti_50 with a porosity of 43.91% and a pore size around 340 μm) and considerable compressive strength could be obtained through the current process. Cell proliferation (MTS) and lactate dehydrogenase (LDH) assays showed that all P_Ti samples exhibited high cell affinity and low cell mortality, indicating good biocompatibility. Among them, P_Ti_50 showed relatively good in-cell morphology and viability, and is thus promising as a load-bearable bio-scaffold.

## 1. Introduction

Bone is an open-cell composite material composed of a complex vascular system and protein-related materials. At the architectural level, bone is made up of two types of tissue tightly packed together. The outer shell is made of dense compact or cortical bone, and the inner core comprises porous cellular, cancellous, or trabecular bone. Cortical bone is highly dense and contains cylindrically organized osteons, whose size is in the range of 10–500 μm [1]. Blood vessels are interconnected on the surface of bone through perforating canals. Unlike cortical bone, cancellous bone is highly porous, consisting of an interconnected network of trabeculae, which are about 50–300 μm in diameter. These two types of bone tissue differ in porosity or density. The porosity of cortical bone is 5–10%, and that of cancellous bone is 75–90% [2,3,4].

Biomimetic (bio-)scaffolds have been increasingly utilized to repair or rebuild osteoporosis defects [5,6,7]. Bone defects are usually caused by trauma or tumors, and bone resorption is initiated by infection. Many types of osseous reconstructive surgical procedures have been studied [8]. However, the three-dimensional properties of bone tissues are required to guide bone-forming cells and the subsequent successful integration of the bone-grafting material in the host tissue. The main reasons for using bone scaffolds are thus to provide an environment for bone formation, maintain space, and provide mechanical support to the bone skeleton during the repair process [4,6,9,10].

Porous ceramic and polymer biomaterials are usually unsuitable for implanting at load-bearing sites. Plasma spraying [11], space holders [12], common powder metallurgy (P/M) [13], and sintered metal-based fibers [14] have been applied for refilling bone defects (e.g., cavities) and regenerating soft tissues. However, it is still difficult to produce a porous structure with an architecture that meets both osteoconductive and mechanical requirements. Porous titanium (P_Ti)-based scaffolds are promising since they may have superior mechanical properties with high strength/weight ratios. It is imperative for dental and orthopaedic surgeons to have a basic understanding of the process of peri-implant bone formation [15,16,17].

The development of open porous structures has been hampered by limitations of production techniques. For osteoconduction, an open interconnected porous structure with pore sizes in the range of 200–500 μm is estimated to be required, though there is no consensus regarding the perfect size of pores for stimulating cell proliferation [18]. From a mechanical point of view, the porous structure should be stiff enough to sustain physiological loads, but should not drastically exceed the stiffness of the bone being replaced to avoid stress shielding. Implant fixation to the bone surface or matrix should be improved through alternatives for reducing stress shielding, which is a consequence of the mismatch between Young’s modulus values (e.g., 110 GPa for solid Ti and 14–20 GPa for cortical bone) [1]. This difference has been identified as one of the major reasons leading to implant loosening and bone resorption. Although Ti and its alloy (Ti-6Al-4V) [6,19,20,21] have the lowest elastic modulus of metallic biomaterials (e.g., 50% lower than that of Co-Cr-based alloys), divergence with respect to bone stiffness remains a challenging problem. Manufacturing implants with lower-bulk-stiffness materials may be a solution for stress shielding.

The development of P_Ti-based scaffolds is a promising approach for achieving stiffness reduction [22,23,24]. However, an important issue for the use of porous materials for load-bearing applications is the potential risk of reducing both mechanical strength and fatigue resistance [12]. Therefore, balancing strength and stiffness is probably the most vital challenge to this approach. Many different formulations, in terms of the material constituents, fabrication technologies, as well as structural and bioactive properties have been proposed [25]. The nano-structurally controlled biocomposites, which contain biomimetically fabricated formulations with collagen, chitin, and/or gelatin, can be considered for the structures of scaffolds [25]. In addition, a scaffold made of nanofibers is competent to provide a structural support for cells to accommodate and guide their growth in a three-dimensional network into a specific tissue [26]. Recently, extensive research has been conducted on fabricating open-cell P_Ti scaffolds, especially through the P/M route [12,27]. This is attributed to the fact that Ti scaffolds are much more difficult to process in the liquid state due to their very high melting temperature (1670 °C). Another problem is their extreme chemical affinity to atmospheric gases (i.e., O_2_ and N_2_), which dissolve rapidly either in liquid or solid Ti at a temperature of above 400 °C [28,29]. Several methods have been applied for producing open-cell Ti foams. The P/M method with a temporary (or removable) space holder, including carbamide, ammonium bicarbonate, tapioca starch, sodium chloride, and magnesium [22,30,31,32,33,34,35], is very attractive for fabricating highly porous samples. These temporary space holders were primarily selected on the basis of various criteria, such as reactivity with Ti, ease of residue removal, and ease of production. The P/M method offers many advantages, such as the adjustment of porosity fraction and pore shape, size, and distribution, depending on the shape, size, and volume fraction of the space holder used for preparing P_Ti samples [12,24]. For medical applications, NaCl may be a good temporary space holder since its residue can be easily removed through dissolution in water [6,33]. As a consequence, P_Ti samples with different forms [36] or ratios [37,38] of porosity can be manipulated by the adjustment of NaCl content in Ti powder, followed by a hydrothermal process.

Compared with a typical process used for preparing P_Ti samples, the hydrothermal method is suitable for removing the temporary space holder. In this study, an enhanced hydrothermal method with high temperature and high pressure is applied for materials processing to prepare load-bearable P_T bio-scaffolds, followed by the assessments of load-bearing capability and biocompatibility.

## 2. Experimental Section

### 2.1. Preparation of Porous Ti Samples

**Figure 1:**
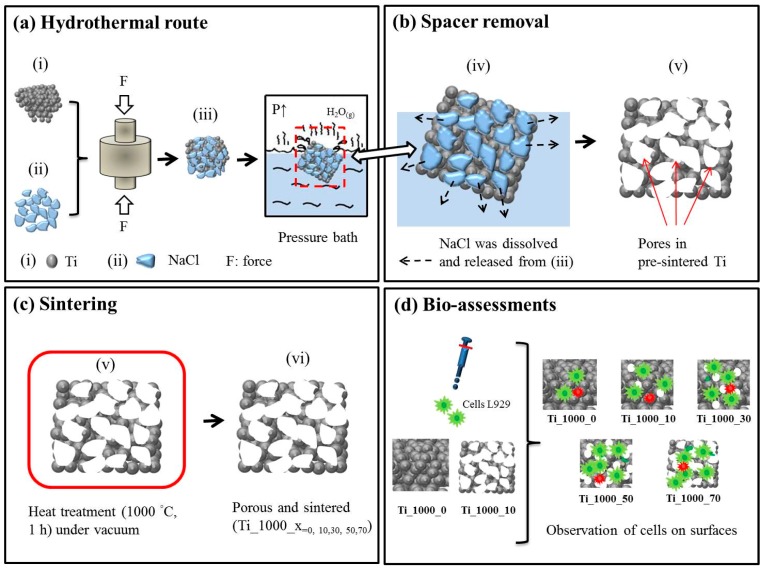
Fabrication processes. (**a**) Hydrothermal route; (**b**) spacer removal; (**c**) sintering; and (**d**) bio-assessments.

Porous Ti samples with a particle size of 45 μm (Zhongrui Material Technology Corp., Tainan, Taiwan) were prepared via the P/M method using NaCl (Taiyen Biotech Corp., Tainan, Taiwan) with a particle size in the range of 180–300 μm as the space holder. In the experiment, Ti and NaCl powders are mixed in wt %. The mixed powders are put inside a 250-mL bottle with 150 mL alcohol. No other binder or reagent is added. The rotating speed of the ball mixer is 300 rpm/min for 24 h. Then, the alcohol is evaporated by putting the bottle in the oven for another 24 h. The purpose of using the hydrothermal method is to remove the space holder, NaCl, from the porous Ti and to form a porous structure. Figure 1a shows the fabrication process of the samples. NaCl was used as a temporary space holder because of its high solubility in water (359 g/L at room temperature), complete inertness with Ti powder, and very low toxicity. Mixtures containing Ti powder and 0–70 wt % NaCl (with respect to the weight of Ti powder) were compacted into a cylindrical disc, 10 mm in diameter and 7 mm in thickness. The compression stress is 300 MPa. In Figure 1b, the as-formed Ti and P-Ti samples were placed into an autoclave, which was also the container for the hydrothermal process. To remove NaCl completely before sintering, the applied temperature was higher than 130 °C to transform the water into water vapour. The as-heated Ti samples were dried at 60 °C in a cyclic oven (JA-27, Great Tide Instrument, Taipei, Taiwan) for 24 h to evaporate the remaining water. As shown in Figure 1c, the solid Ti sample is evacuated three times under vacuum (10^−3^ mbar), filled with argon gas (to avoid Ti oxidation), and then sintered at 1000 °C for 2 h. The as-sintered solid Ti sample is denoted as Ti_1000_0. Using the same process, Ti samples with 10, 30, 50, and 70 wt % additions of NaCl were prepared. The as-sintered “porous” (i.e., after NaCl removal) Ti samples are denoted as Ti_1000_10, _30, _50, and _70, respectively. The samples were stored for further studies, e.g., bio-assessments as shown in Figure 1d.

### 2.2. Surface Characterization

The physical and chemical properties of the as-prepared porous Ti samples were characterized. To verify the residue of NaCl in Ti samples, inductively coupled plasma mass spectrometry (ICP-MS; Thermo Element XR, Waltham, MA, USA) was employed. This test was performed according to the standard ISO-10993. The crystalline structure of P_Ti samples after hydrothermal treatment was determined using X-ray diffraction (XRD; MiniFlex II, Rigaku, Tokyo, Japan) with CuKα radiation. The surface morphology of the as-prepared P_Ti samples was examined using a Field Emission Scanning Electron Microscope (FE-SEM, JSM-7001, JEOL, Tokyo, Japan). The Ti and P_Ti samples were sputter-coated with a layer of Pt and then observed under a normal condition (i.e., with an accelerating voltage of 10 kV and under a chamber vacuum of 4.13 × 10^−3^ Pa) using an optical microscope (Neophoto-32, ZEISS, Oberkochen, Germany). The resulting porosity measurement was carried out using the Archimedes method (with distilled water) due to its experimental simplicity and reasonable reliability (ASTM C373-88) [39].

### 2.3. Compression and Nanoindentation Tests

For the compression test, the sample’s dimension was fixed according to the standard ISO-5833 [40,41]. The yield strength and relative strength (defined as the ratio of the strength of the porous material to that of the solid material) were then obtained.

The nanomechanical properties were measured using a continuous stiffness measurement (CSM) system (Nano Indenter XP, MTS, Palo Alto, CA, USA), which produces highly sensitive load-displacement data at the surface contact level. In the experiment, the triangular pyramid tip of a Berkovich diamond with a radius of ≈20 nm was used under a controlled relative humidity of 45% at 22 °C. Poisson’s ratio for the tested porous Ti samples was set to 0.32. The loading process was controlled to have a surface approach velocity of 1 nm/s with a sensitivity of 5%. A constant strain rate of 0.05/s at a chosen frequency of 75 Hz was applied. The calculation of nano-hardness was mainly based on the Oliver and Pharr method.

### 2.4. In Vitro Tests

Figure 1d shows the in vitro assessment for the as-prepared porous Ti samples. According to the standard ISO-10993, the live/dead L929 cell staining protocol, cell proliferation (MTS) assay, and lactate dehydrogenase (LDH) assay were respectively employed. Earlier toxicological studies used similar cell lines to provide a basis for comparison [42,43,44,45]. The mean cell culture activity provides an assessment of the cells’ overall activity, which is an indicator of stress, toxic effects targeting metabolic pathways, and overall viability. Fibroblast cells derived from an immortalized mouse fibroblast cell line were preserved in alpha modified Eagle’s medium (α-MEM) with 10% horse serum (Gibco, Invitrogen, Carlsbad, CA, USA) and 10 mL of 10^4^ units/mL penicillin −10^4^ μg/mL streptomycin (Sigma, St. Louis, MO, USA). Before the experiments, fibroblast cells were washed with phosphate-buffered saline (PBS) and detached with trypsine (Gibco, Invitrogen). For the MTS assay, the fibroblast cells were then cultured in a complete medium maintained at 37 °C in a 5% CO_2_ incubator for 24 h, attained to 7.5 × 10^5^ cells/mL in a complete medium, and again maintained at 37 °C under 5% CO_2_ for 24 h. For the MTS and LDH assays, the fibroblast cells were seeded near confluence (2 × 10^4^ cells/well = 6.75 × 10^5^ cells/mL) on 24-well plates (Nunc, Thermal Scientific, Rochester, NY, USA).

## 3. Results and Discussion

### 3.1. Composition of As-Prepared Porous Ti Samples

**Figure 2:**
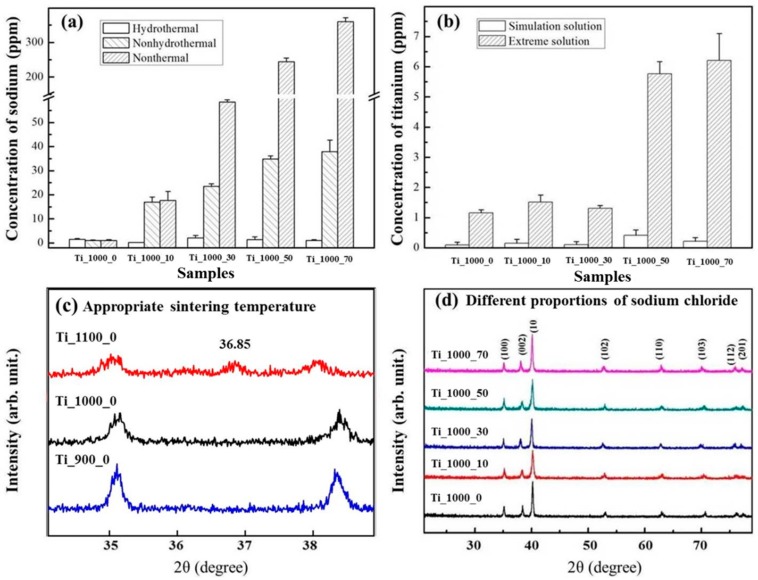
The ICP-MS measurements and XRD patterns of porous Ti samples. (**a**) ICP-MS spectrum for Na; (**b**) results of simulation and extreme solution test; (**c**) XRD patterns of pure Ti sintered at 900–1100 °C; and (**d**) XRD patterns of samples with various NaCl concentrations sintered at 1000 °C.

The ICP-MS measurements and XRD patterns of Ti and P_Ti samples are shown in Figure 2a–d, respectively. In Figure 2a, the result of ICP-MS measured data for Na concentration is shown. As compared to the traditional P/M process to form a P_Ti, Na concentrations in the modified ones for Ti_1000_10, _30, _50, and _70 were higher (e.g., Ti_1000_70 contained Na residue of up to 336 ppm). After the hydrothermal process, the Na concentration decreased to less than 3 ppm. The hydrothermal process is thus capable of removing Na for preparing P_Ti samples. Figure 2b shows that under the simulation and extreme solution test based on ISO-5833, trace Na concentrations of 5.7 ppm (Ti_1000_50) and 6.2 ppm (Ti_1000_70) were achieved.

The characteristics of the Ti structure after heat treatment at 900–1100 °C were examined using XRD. Figure 2c,d show that with a heat treatment at around 1100 °C, a new diffraction peak appeared at 2θ = 36.85°, which shows that Ti was most probably oxidized at above 1000 °C. Figure 2d shows the XRD patterns of the as-prepared Ti samples with various added percentages of NaCl after annealing at up to 1000 °C. The peaks at 2θ = 25° and ~77.3°, which appeared at 1000 °C, are respectively assigned to the (100), (002), (101), (102), (110), (103), (112), and (201) reflections of tetragonal Ti (JCPDS card No. 44-1294). The diffraction peaks were the same for all samples. The results show that the addition of NaCl did not cause any change in the characteristics of the Ti structure.

**Figure 3:**
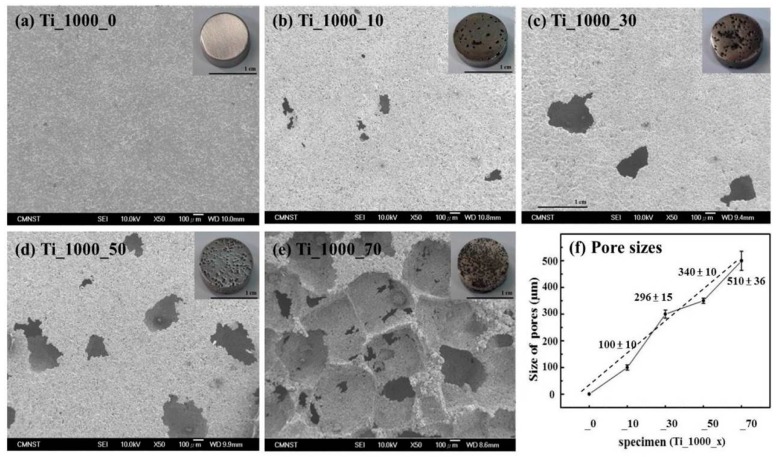
SEM images of (**a**) Ti_1000_0; (**b**) Ti_1000_10; (**c**) Ti_1000_30; (**d**) Ti_1000_50; and (**e**) Ti_1000_70; (**f**) Corresponding pore sizes.

SEM micrographs and pore sizes of the P_Ti samples are shown in Figure 3a–f. The pore sizes of P_Ti samples ranged from 100 to 500 μm; this range has been shown to allow cell ingrowth [4]. Moreover, the connectivity of the porous network structure, created by the removal of NaCl from the Ti matrix, was presumably facilitated by the hydrothermal process. As shown in Figure 3b–e, the pore sizes of Ti_1000_10, _30, _50, and _70 were estimated to be 100 ± 10, 296 ± 15, 340 ± 10, and 500 ± 36 μm, respectively. As shown in Figure 3f, the samples Ti_1000_30 and _50 were particularly suitable for subsequent cell ingrowth, most probably because of their appropriate pore sizes.

### 3.2. Load-Bearing Capacity of As-Prepared Porous Ti Samples

**Figure 4:**
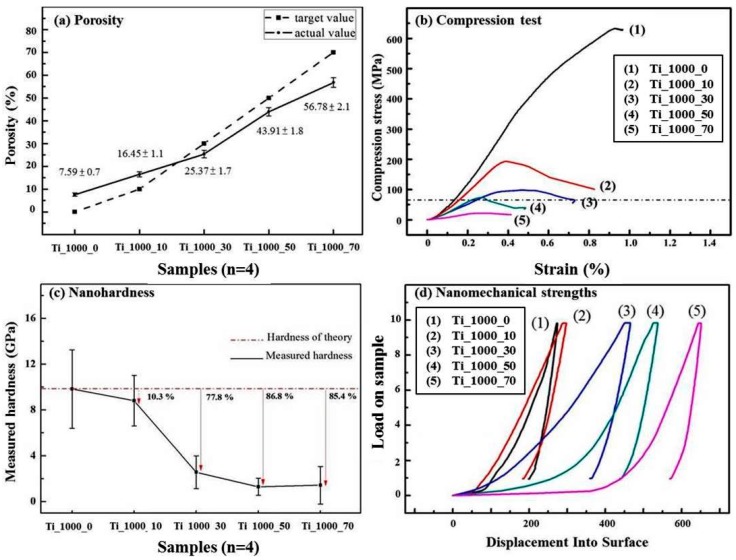
Quality and compressive strength of porous Ti samples. (**a**) Porosity; (**b**) compressive strength; (**c**) nano-hardness; and (**d**) nanomechanical strength.

Figure 4a–d show the results of porosity, compressive strength, and nano-hardness for the P_Ti samples. As shown in Figure 4a, the porosities of Ti_1000_10, _30, _50, and _70 were 16.45 ± 1.1, 25.37 ± 1.7, 43.91 ± 1.8, and 56.78 ± 1.2%, respectively, as compared with 7.59 ± 0.9% for Ti_1000_0. The added percentages of NaCl slightly correspond to the measured porosities for relatively low added percentages.

In Figure 4b it is shown that the measured values for compressive strength were 633, 182, 97, 73, and 23 MPa for Ti_1000_0, _10, _30, _50, and _70, respectively. The resistance to a given compression force decreased with increasing porosity of the P_Ti samples. According to ISO 5833, for an implantable and load-bearing scaffold, a compressive strength for the bulk material should be higher than 70 MPa. Therefore, Ti_1000_70 does not meet the required strength.

Figure 4d shows that the nano-hardness values obtained using a nano-indentator for Ti_1000_0, _10, _30, _50, and _70 were 9.8 ± 3.4, 8.1 ± 2.2, 2.6 ± 1.6, 1.3 ± 0.5, and 1.4 ± 0.6 GPa, respectively. Note that since the test is usually focused on the solid part of the porous support, the measured nano-hardness value does not fully reflect the real pore condition in the P_Ti sample. In spite of this, the results imply that the porosity varied with the measured nano-hardness and thus the Ti scaffold structure is presumably consistent with the density of pores.

From this study, it is therefore promising to obtain a load-bearable P_Ti scaffold with a highly porous structure, e.g., P_Ti_50 with the porosity of 43.91 ± 1.8%, and a considerable compressive strength higher than 70 MPa.

### 3.3. Cell Affinity of Porous Ti Samples

**Figure 5:**
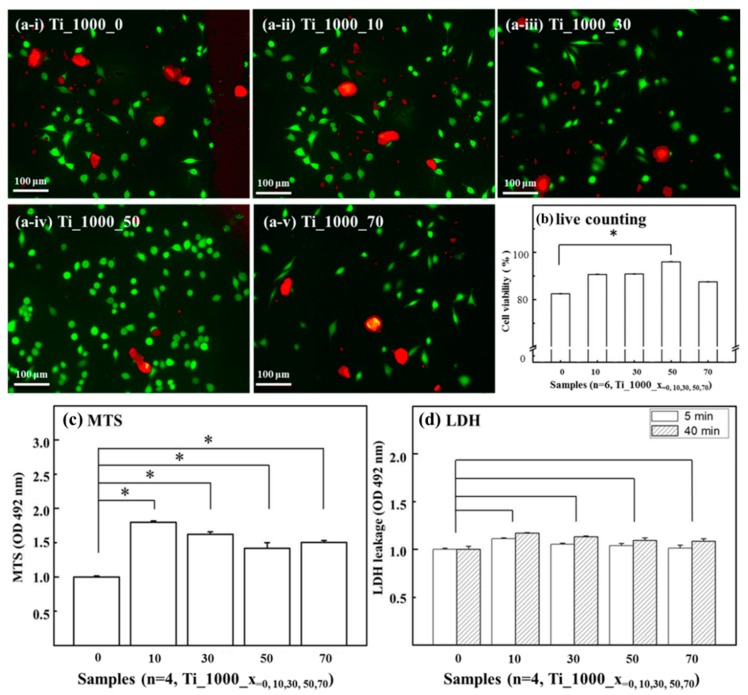
Optical microscopy images for live/dead staining protocol on surfaces of (**a**-**i**) Ti_1000_0; (**a**-**ii**) Ti_1000_10; (**a**-**iii**) Ti_1000_30; (**a**-**iv**) Ti_1000_50; and (**a**-**v**) Ti_1000_70; (**b**) Summary of cell viability for these surfaces. (**c**) Results of the cell proliferation MTS assay; (**d**) Effects of lactate dehydrogenase LDH in early stage for Ti_1000_0, Ti_1000_10, Ti_1000_30, Ti_1000_50, and Ti_1000_70. No significant differences in LDH level on surfaces were found between samples.

By taking Ti_1000_0 as the reference substrate, cell morphology and viability were assessed for the P_Ti samples. Figure 5a (i)–(v) shows optical microscopy images for the live/dead staining protocol. A comparison of cell viability for the samples is shown in Figure 5b. Significant enhancements in fibroblast cell attachment and viability were found for Ti_1000_50.

Figure 5c shows the MTS assay results for the P_Ti samples. By taking Ti_1000_0 as the control group, significant differences (*p* < 0.05) were found for all porous samples. This indicates that a P_Ti structure, regardless of its porosity, tends to enhance its cell viability.

Figure 5d shows the levels of LDH leakage for the P_Ti samples. Note that the testing cells are presumably affected by the composition of the contact surface, which may lead to lipid peroxidation and sub-lethal effects on the membranes of the cells; the effects of LDH leakage are a result of the formation of pores in the cell membrane. The results show that, at an early stage of the LDH leakage test, there was no significant difference in the LDH level between the surface of Ti_1000_0 and those of Ti_1000_10, _30, _50, and _70.

From the above tests, it can be seen that the surface composition and structure of the P_Ti samples exhibited biocompatibility; in particular, the sample Ti_1000_50 showed relatively high cell affinity.

### 3.4. Comparison with Commercially Available Porous Ti-Based Scaffolds

**Figure 6:**
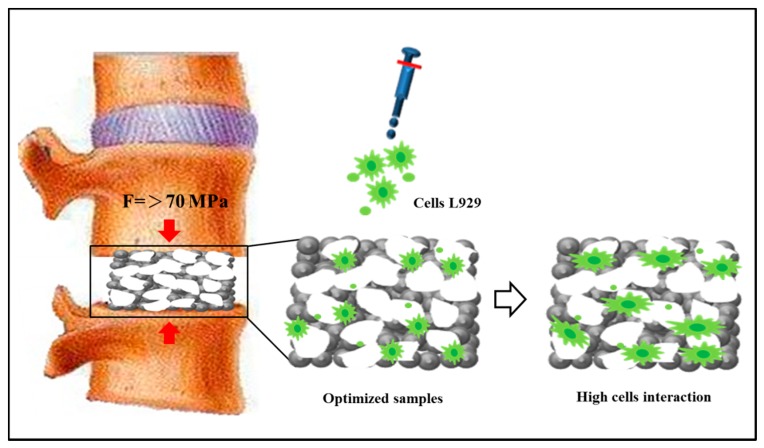
Porous Ti-based scaffold with high biocompatibility.

Figure 6 shows an ideal P_Ti-based sample. As mentioned, an implantable and load-bearing scaffold should have a compressive strength of higher than 70 MPa. In this study, the sample Ti_1000_50 had a compressive strength of 73 MPa and a measured porosity of 43.91 ± 1.8%. Moreover, all the porous samples showed relatively high cell affinity with excellent cell growth on the surfaces and inside the pores, as compared to that for the solid Ti sample. The pore size of Ti_1000_50 was measured to be around 340 ± 10 μm, which is comparable to that of human trabecular bone. A comparison with commercially available porous scaffolds is listed in Table 1. The load-bearing capacity, porosity, pore size, and biocompatibility of Ti_1000_50 make it suitable as a replacing structure for e.g., the lumbar disc of the spine or a part of trabecular bone.

## 4. Conclusions

In this study, P_Ti samples were fabricated by mixing Ti powder with NaCl, followed by a hydrothermal process, which removed NaCl from the P_Ti matrix and created solid pore sites. An annealing temperature of 1000 °C was optimal for preparing P_Ti samples without altering the lattice structure of Ti. With an increased weight percentage of NaCl, the pore size as well as the porosity increased. By comparing the porosity with the required compressive strength of P-Ti bulk and biocompatibility on the P_Ti samples, the sample Ti_1000_50 is thus the most appropriate load-bearable bio-scaffold for e.g., human trabecular bone.

## Figures and Tables

**Table 1:** Comparison with commercially available porous scaffolds (experimental group/Zimmer: TM-S /BAUI: 848-05133).

Sample	Pore Size (μm)	Porosity (%)	Mechanical Properties (MPa)	Biocompatibility
Ti_1000_50	340 ± 10	43.91 ± 1.8	73	excellent
M-S	>300	80	-	excellent
848-05133	<200	-	-	excellent

## References

[1] Nouri A., Hodgson P.D., Wen C.E. (2010). Biomimetic Porous Titanium Scaffolds for Orthopaedic and Dental Applications.

[2] Burr D.B., Martin R.B. (1989). Errors in bone remodeling: Toward a unified theory of metabolic bone disease. Am. J. Anat..

[3] Choi K., Kuhn J.L., Ciarelli M.J., Goldstein S.A. (1990). The elastic moduli of human subchondral, trabecular, and cortical bone tissue and the size-dependency of cortical bone modulus. J. Biomech..

[4] Rho J.Y., Kuhn-Spearing L., Zioupos P. (1998). Mechanical properties and the hierarchical structure of bone. Med. Eng. Phys..

[5] Rajangam T., An S.S.A. (2013). Fibrinogen and fibrin based micro and nano scaffolds incorporated with drugs, proteins, cells and genes for therapeutic biomedical applications. Int. J. Nanomed..

[6] Bansiddhi A., Sargeant T.D., Stupp S.I., Dunand D.C. (2008). Porous NiTi for bone implants: A review. Acta Biomater..

[7] Imwinkelried T. (2007). Mechanical properties of open-pore titanium foam. J. Biomed. Mater. Res. Part A.

[8] Guerrier G., Alaqeeli A., Al Jawadi A., Foote N., Baron E., Albustanji A. (2015). Reconstruction of residual mandibular defects by iliac crest bone graft in war-wounded Iraqi civilians, 2006–2011. Br. J. Oral Maxillofac. Surg..

[9] Silva G.A., Coutinho O.P., Ducheyne P., Reis R.L. (2007). Materials in particulate form for tissue engineering. J. Tissue Eng. Regen. Med..

[10] Kumar A., Mandal S., Barui S., Vasireddi R., Gbureck U., Gelinsky M., Basu B. (2016). Low temperature additive manufacturing of three dimensional scaffolds for bone-tissue engineering applications: Processing related challenges and property assessment. Mater. Sci. Eng. R Rep..

[11] Jaeggi C., Frauchiger V., Eitel F., Stiefel M., Schmotzer H., Siegmann S. (2011). The effect of surface alloying of Ti powder for vacuum plasma spraying of open porous titanium coatings. Acta Mater..

[12] Li B.Q., Wang C.Y., Lu X. (2013). Effect of pore structure on the compressive property of porous Ti produced by powder metallurgy technique. Mater. Des..

[13] Kim S.W., Jung H.D., Kang M.H., Kim H.E., Koh Y.H., Estrin Y. (2013). Fabrication of porous titanium scaffold with controlled porous structure and net-shape using magnesium as spacer. Mater. Sci. Eng. C Mater. Biol. Appl..

[14] Zou C., Zhang E., Li M., Zeng S. (2008). Preparation, microstructure and mechanical properties of porous titanium sintered by Ti fibres. J. Mater. Sci. Mater. Med..

[15] Abbah S.A., Lam C.X., Hutmacher D.W., Goh J.C., Wong H.K. (2009). Biological performance of a polycaprolactone-based scaffold used as fusion cage device in a large animal model of spinal reconstructive surgery. Biomaterials.

[16] Youm I., Youan B.B.C. (2013). Uptake mechanism of furosemide-loaded pegylated nanoparticles by cochlear cell lines. Hear. Res..

[17] Haugen H.J., Monjo M., Rubert M., Verket A., Lyngstadaas S.P., Ellingsen J.E., Wohlfahrt J.C. (2013). Porous ceramic titanium dioxide scaffolds promote bone formation in rabbit peri-implant cortical defect model. Acta Biomater..

[18] Wen C.E., Mabuchi M., Yamada Y., Shimojima K., Chino Y., Asahina T.T. (2001). Processing of biocompatible porous Ti and Mg. Scr. Mater..

[19] Rahmati B., Sarhan A.A., Basirun W.J., Abas W.A.B.W. (2016). Ceramic tantalum oxide thin film coating to enhance the corrosion and wear characteristics of Ti 6Al 4V alloy. J. Alloys Compd..

[20] Ribeiro Filho S.L.M., Lauro C.H., Bueno A.H.S., Brandão L.C. (2016). Influence cutting parameters on the surface quality and corrosion behavior of Ti-6Al-4V alloy in synthetic body environment (SBF) using Response Surface Method. Measurement.

[21] Rahmati B., Sarhan A.A., Zalnezhad E., Kamiab Z., Dabbagh A., Choudhury D., Abas W.A.B.W. (2016). Development of tantalum oxide (Ta-O) thin film coating on biomedical Ti-6Al-4V alloy to enhance mechanical properties and biocompatibility. Ceram. Int..

[22] Ye B., Dunand D.C. (2010). Titanium foams produced by solid-state replication of NaCl powders. Mater. Sci. Eng. A-Struct. Mater. Prop. Microstruct. Process..

[23] Torres Y., Lascano S., Bris J., Pavón J., Rodriguez J.A. (2014). Development of porous titanium for biomedical applications: A comparison between loose sintering and space-holder techniques. Mater. Sci. Eng. C Mater. Biol. Appl..

[24] Jha N., Mondal D.P., Majumdar J.D., Badkul A., Jha A.K., Khare A.K. (2013). Highly porous open cell Ti-foam using NaCl as temporary space holder through powder metallurgy route. Mater. Des..

[25] Dorozhkin S.V. (2011). Biocomposites and hybrid biomaterials based on calcium orthophosphates. Biomatter.

[26] Murugan R., Ramakrishna S. (2007). Design strategies of tissue engineering scaffolds with controlled fiber orientation. Tissue Eng..

[27] Wang X.H., Li J.S., Hu R., Kou H.C., Zhou L. (2013). Mechanical properties of porous titanium with different distributions of pore size. Trans. Nonferr. Met. Soc. China.

[28] Guleryuz H., Cimenoglu H. (2009). Oxidation of Ti-6Al-4V alloy. J. Alloys Compd..

[29] Oshida Y. (2010). Bioscience and Bioengineering of Titanium Materials.

[30] Arifvianto B., Leeflang M.A., Zhou J. (2015). The compression behaviors of titanium/carbamide powder mixtures in the preparation of biomedical titanium scaffolds with the space holder method. Powder Technol..

[31] Aydoğmuş T., Bor Ş. (2009). Processing of porous TiNi alloys using magnesium as space holder. J. Alloys Compd..

[32] Mansourighasri A., Muhamad N., Sulong A.B. (2012). Processing titanium foams using tapioca starch as a space holder. J. Mater. Process. Technol..

[33] Torres Y., Pavón J.J., Rodríguez J.A. (2012). Processing and characterization of porous titanium for implants by using NaCl as space holder. J. Mater. Process. Technol..

[34] Esen Z., Bor Ş. (2007). Processing of titanium foams using magnesium spacer particles. Scr. Mater..

[35] Zhang X., Li X.W., Li J.G., Sun X.D. (2014). Preparation and mechanical property of a novel 3D porous magnesium scaffold for bone tissue engineering. Mater. Sci. Eng. C Mater. Biol. Appl..

[36] Caparrós C., Ortiz-Hernandez M., Molmeneu M., Punset M., Calero J.A., Aparicio C., Gil F.J. (2016). Bioactive macroporous titanium implants highly interconnected. J. Mater. Sci. Mater. Med..

[37] Chen L.J., Ting L.I., Li Y.M., Hao H.E., Hu Y.H. (2009). Porous titanium implants fabricated by metal injection molding. Trans. Nonferr. Met. Soc. China.

[38] Torres Y., Trueba P., Pavón J.J., Chicardi E., Kamm P., García-Moreno F., Rodríguez-Ortiz J.A. (2016). Design, processing and characterization of titanium with radial graded porosity for bone implants. Mater. Des..

[39] Jin X., Dong L., Xu H., Liu L., Li N., Zhang X., Han J. (2016). Effects of porosity and pore size on mechanical and thermal properties as well as thermal shock fracture resistance of porous ZrB_2_–SiC ceramics. Ceram. Int..

[40] Cimatti B., Engel E.E., Nogueira-Barbosa M.H., Frighetto P.D., Volpon J.B. (2015). Physical and mechanical characterization of a porous cement for metaphyseal bone repair. Acta Ortop. Bras..

[41] Van de Graaf G.M.M., Zoppa D., do Valle A.L., Moreira R.C., Maestrelli S.C., Marques R.F.C., Campos M.G.N. (2015). Morphological and mechanical characterization of chitosan-calcium phosphate composites for potential application as bone-graft substitutes. Res. Biomed. Eng..

[42] Neacsu P., Gordin D.M., Mitran V., Gloriant T., Costache M., Cimpean A. (2015). In vitro performance assessment of new beta Ti–Mo–Nb alloy compositions. Mater. Sci. Eng. C Mater. Biol. Appl..

[43] Hiromoto S., Hanawa T., Asami K. (2004). Composition of surface oxide film of titanium with culturing murine fibroblasts L929. Biomaterials.

[44] Tianshi W., Renji Z., Yongnian Y. (2009). Preparation of bioactive hydroxyapatite on pure titanium. J. Bioact. Compat. Polym..

[45] Zhang E., Zou C., Yu G. (2009). Surface microstructure and cell biocompatibility of silicon-substituted hydroxyapatite coating on titanium substrate prepared by a biomimetic process. Mater. Sci. Eng. C Mater. Biol. Appl..

